# Parental knowledge and attitude of postoperative paediatric pain: stepwise linear regression analysis

**DOI:** 10.3389/fpain.2024.1340375

**Published:** 2024-03-19

**Authors:** Mitiku Desalegn, Tewoderos Shitemaw, Genanew Kassie Getahun, Lire Lemma

**Affiliations:** ^1^ Department of Anesthesia, College of Medicine and Health Science, Wachemo University, Hossana, Ethiopia; ^2^ Menelik II Medical and Health Science College, Addis Ababa, Ethiopia; ^3^ Department of Public Health, Menelik II Medical and Health Science College, Addis Ababa, Ethiopia; ^4^ Department of Health Informatics, College of Medicine and Health Science, Wachemo University, Hossana, Ethiopia

**Keywords:** parental, knowledge, attitude, pain, medications, linear regression

## Abstract

**Background:**

Despite the fact that mothers care for their children's pain in most cases, it has been noted that mothers have limited knowledge and attitude about paediatric pain. This study aims to assess parental knowledge and attitude of postoperative paediatric pain (POPP).

**Method:**

This is institutional based cross sectional study conducted with 102 parents at Nigist Eleni Mohamed Memorial Comprehensive Specialized Hospital (NEMMCSH). A convenience sampling technique was used to select parents. This study has used a questionnaire (Parental Pain Expression Perception (PPEM), examine parents' attitudes and knowledge about how their children exhibit their pain and Medication Attitude Questioner (MAQs), focuses on how parents feel about giving their child analgesic medication to alleviate post-operative pain). Descriptive statistics were utilized to analyse the parent's response and presented with frequency and percentage. Factor analysis to analyze factor structure and stepwise linear regression analysis to examine the impact of socio-demographic factors in predicting parental knowledge and attitude about POPP were done. The statistical tests were performed at 95% confidence interval and 5% significance level.

**Result:**

A total of 102 parents fulfilling the inclusion criteria were included. About 78% of parents agreed that children always express pain by crying or whining. The majority of parents (75.6%) believe children who are playing are not in pain. Regarding parental attitudes about pain medications, majority of parents (61%) believe that children should be given pain medication as little as possible because of its side effects. According to about 26.8% of parents, giving children pain medication for pain might teach them to use drugs for other issues. On the other hand, 63.4% of parents say that giving children pain medication as little as possible is the most effective way to manage their pain. Parents of younger children and parents from rural area are more likely to score higher in attention seeking sub-score of PPEP while parents from urban residence and those parents who are employed are more likely to perceive about the side effects of pain medications (Side effects factors).

**Conclusion:**

The overall knowledge and attitude of parents about postoperative pain and pain medications were poor.

## Background

The Subcommittee on Taxonomy made the present definition of pain, which was approved by the IASP Council in 1979 and is as follows: An unpleasant sensory and emotional experience connected with existing or potential tissue damage, or defined in terms of such harm (1). In the medical field, it was believed for a long time that children do not feel pain. This misunderstanding was founded on the assumption that neonates, in particular, have immature nervous systems and that neural pathways must fully myelinate to be able to function (2). According to research primarily conducted in high-income countries (HICs), 20%–35% of adolescents and youngsters experience pain (3).

Postoperative pain is defined as the result of tissue damage and postoperative muscle spasm. It is relatively common for acute post-operative pain to progress to chronic pain, with estimates ranging from 5% to 60% (4, 5). Variability in estimates is likely, in part, due to the nature of the surgery and condition. Since poorly managed pain in children can have negative effects, pain management is crucial for positive post-operative results. Early postoperative pain exposure has been linked to a variety of undesirable behavioural and physiological outcomes (6). Acute postoperative pain that is not adequately managed is linked to higher morbidity, functional and quality-of-life impairment, delayed recovery, longer opiate use, and higher healthcare expenditures. Furthermore, the presence and degree of acute pain during or immediately following surgery is a predictor of the development of chronic pain (7).

In light of the important role that parents commonly have in managing children's post-operative pain once they are discharged from the hospital, an understanding of parental knowledge and attitudes regarding children's post-operative pain is valuable. Parental attitudes/knowledge to pain has been studied in various countries and settings. A descriptive cross sectional study from China showed parents have low to moderate level of knowledge and attitude about postoperative children's pain management and use of pain relief strategies (8). Another study from public hospital in Singapore demonstrates that parents have a moderate level of knowledge and attitude about their children's postoperative pain and suggests the need to provide parents with more information regarding their children's pain (9). However, a study conducted in the UK reported that many parents have a good understanding of how children express pain, even though still substantial proportion of parents hold misconceptions regarding how children express pain (10). Little research has been conducted exploring parental attitudes to children's pain in African populations, evidence from two Botswana referral hospitals stated parents have adequate knowledge and positive attitudes about acute pain management among hospitalised children (11).

The focus of research studies, however, has recently switched to parents, especially mothers managing their children's pain at home. This is due to the growing realization that parents tend to address a larger portion of children's suffering at home (12). Despite the fact that parents care for their children's pain in most cases, it has been noted they have limited knowledge, attitude and practice regarding pharmacologic and non-pharmacologic pain management (10, 13).

Even though Paediatric pain assessment is challenging, it is crucial for parents to be able to accurately and efficiently assess and manage their child's pain at home (14). A significant proportion of parents hold erroneous attitude toward postoperative pharmacologic pain management in fear of side effects (10). A lack of knowledge about pain, analgesics, and the advantages and safety of analgesics may be the cause of parents' unwillingness to let their kids take medications (15). Considering the importance of a mother's role and the scarcity of research regarding their knowledge and attitudes towards postoperative pain assessment and management, this study aims to assess parental perception of knowledge and attitude of postoperative paediatric pain.

## Materials and methods

### Study design and setting

A prospective, cross-sectional study was conducted at Wachemo University NEMMCSH from August 1 to October 30, 2022, in Ethiopia. The study involved parents who have children aged between 2 and 12 years undergoing elective surgical procedures. The inclusion criteria included the ability of the legal parents to listen, speak, write, and read in the local language and parents who did not have any diagnosed visual, auditory, sensual, or sensory diseases and had the cognitive competence to answer questions. Three parents were excluded from the study (2 parents were unable to understand the local language and 1 parent was unable to effectively communicate due to a medical condition). In the study, the independent observer was the researcher himself, who was a master's anesthetist with three years of clinical experience working in the operating room and post-anesthesia care unit. A convenience sampling technique was used to select parents.

### Data collection tool, method and procedure

The questionnaire was designed to collect demographic information about the parent and children and items to assess parental knowledge and attitude about pediatric pain and pain medications. The first section had ten questions related to the parental and children's demographics, namely age, marital status, education level, place of residence, monthly income, employment status of parents and age, and sex of children. The second section of the questionnaire consisted of three questions related to surgery (type of procedure, type of anesthesia, and children's previous history of surgical exposure). The third section is related to parental knowledge and attitude towards PPEP, which consists of nine questions to be answered on a 3-point Likert scale (agree, disagree, or uncertain). The fourth section is about MAQ, which consisted of 16 items to be responded to on a 3-point Likert-type scale (agree/disagree/uncertain).

Two final-year health education masters students translated the questionnaire from English into the local language, and two other final-year health education masters students reversed the translation from the local language to English. We contrasted the two translations. No particular issue was found, and nothing was altered. A pilot study was done with 5% of the parents who met the inclusion criteria prior to data collection from target parents. The purpose of the pilot study was to evaluate the validity of the questionnaires. The pilot study is not being reported in the current manuscript. For the purpose of data collection, four final-year anesthesia students were assigned after receiving training about data collection tools, procedures, and inclusion and exclusion criteria. The data collectors were strictly supervised by two senior anesthetists to ensure the completeness of the data.

Parents were approached and recruited on the day of surgery. After informed consent is obtained, a questionnaire containing items with demographics, parental knowledge about pain and pain medication attitude is provided during the preoperative period. Parents were informed that the study aimed to examine parents' knowledge and attitudes towards pain and pain medications, with the aim of informing them about pain management provided to children post-operatively. The data were collected over a period of three months.

### Measures

#### Parental pain expression perceptions (PPEP)

PPEP examine parents' attitudes and knowledge about how their children exhibit their pain. It is composed of nine items, each of which has been given a rating on a 3-point scale (Agree, disagree and uncertain). The PPEP has been shown to have strong content and construct validity (16), and this study's Cronbach's alpha internal consistency was (*a* = 0.79).

#### Medication attitudes questionnaire (MAQ)

MAQ focuses on how parents feel about giving their child analgesic medication to alleviate post-operative pain. The question consists of 16 items, each of which is assessed from three choices (agree, disagree or uncertain). The MAQ has demonstrated good content and predictive and construct validity (17), which was 0.68 in the current study.

### Data analysis

The Statistical Package for Social Sciences (SPSS), version 25, was applied to analyse the data. Descriptive statistics were utilized to analyse socio-demographic variables and frequency responses of parents for PPEP and MAQ items. The data were analysed in terms of individualized items and the number of correct responses for each item. The total score is provided as a percentage of correct answers by dividing the total number of correct responses by the total number of PPEP and MAQ items X/9 and X/16, respectively. Based on Blooms cut-off point, scores above 80% (≥7 correct responses on the PPEM item and ≥13 correct responses on the MAQ item) were taken as good knowledge and good attitude respectively about pain and pain medication in the postoperative period (18). Exploratory factor analysis using maximum likelihood method with Varimax rotation is used for analysing the factor structure and correlation between PPEP and MAQ items. Cronbach's Alpha (α) was used to evaluate the internal consistency of items within each factor. Stepwise linear regression analysis to examine the impact of socio-demographic factors in predicting parental knowledge and attitude about postoperative paediatric pain (POPP) were done.

### Ethical consideration

Permission to conduct the study was obtained from the local research ethics committee. Parents were provided with an information sheet detailing what taking part in the study would involve. Parent's information sheets clearly stated there was no obligation to participate and that not doing so would not affect their child's care. As participants were completing questionnaires, consent was inferred; if they did not wish to participate, they would not complete the questionnaire. No personal data was collected since questionnaires were completed anonymously.

## Result

### Socio demographic characteristic of parents and children

In this study, a total of 105 parents were included with a response rate of 97.1% (102). In total, the number of parents whose ages were between 20 and 29 and 30–39 years was similar (40, 39%). A variety of surgical procedures were performed during the study period including skin graft, foreign body removal, hernia repair, circumcision, repair of tear on eyelid, applying hip spica, wire insertion and removal from bone and others. Almost all parents were married (99, 97%), and the number of parents living in rural (50, 49%) and urban (52, 51%) areas was comparable. Regarding the educational status of study participants, elementary (30, 29.4%), primary school (37, 36.3%), secondary school (32, 31.3%), and university graduates (3, 2.95%). Most of the children were male (82, 80.4%), and their mean age was 6.21 ± 3.3, about 55, 53.9%) of the data were collected from mothers (Table 1).

**Table 1:** Socio demographic characteristic of parents and children (*n* = 102).

Variable	Category	Frequency	Percent
Age (year)	20–29	40	39
	30–39	40	39
	≥40	22	22
Marital status:	Married	99	97
	Divorced	3	3
Education level	Elementary	30	29.4
	Primary school	37	36.3
	Secondary school	32	31.3
	University	3	2.95
Place of residence:	Urban	52	51
	Rural	50	49
Income	Low	42	41.2
	Middle	60	58.8
Status of employment	Employed	42	41.2
	Unemployed	60	58.8
Relationship with child:	Mother	55	54
	Father	45	44.1
	Other	2	1.9
Age of child in year	2–5	45	44.2
	6–12	57	55.8
Gender of child	Male	82	80.4
	Female	20	19.6

### Response of parents for PPEP items

About 78% of parents agreed that children always express pain by crying or whining, whereas 17% of parents disagreed with it, and 5% of them were unsure. Nearly 60% of parents reported that children always tell their parents when they are in pain, but 17% of respondents disagreed with that thought. The majority of parents (75.6%) believed children who were playing were not in pain, although 20% of parents were against that belief. Around 44% of parents consider that children exaggerate pain, and the same number of parents did not support the idea. Most parents (70%) disagreed with the idea that children feel less pain than adults, and the majority of parents (78%) assume children have trouble sleeping when they are in pain (Table 2).

**Table 2:** Response of parents for PPEP items (*n* = 102).

PPEP items	Agree, %	Disagree, %	Uncertain, %
1. Children always express pain by crying or whining	78	17.1	4.9
2. Children always tell their parents when they are in pain	58.5	24.4	17.1
3. Children who are quiet are not in pain	46.3	41.5	12.2
4. Children who are playing are not in pain	75.6	19.5	4.9
5. Children experiencing pain report it immediately	34.1	51.2	14.6
6. Children exaggerate pain	43.9	43.9	12.2
7. Children complain about pain to get attention	58.5	24.4	17.1
8. Children feel pain less than adults	22	65.9	12.2
9. Children in pain have trouble sleeping	78	19.5	2.4

### Response of parents for MAQ items

The majority of parents (61%) believe that children should be given pain medication as little as possible because of its side effects, although around 44% of respondents said there was little need to worry about the side effects. Nearly 26.8% of parents consider that if children are given pain medication, they will learn to use it for other purposes; however, 44% of parents are uncertain about it. 63.4% of parents respond that pain medication works best when it is given as little as possible, and 44% of participants are not sure whether pain medication is addictive. Nearly half of parents (48.8%) stated pain medication has many side effects, and 41.5% of parents are uncertain about whether their children will become addicted to pain medications. Most parents (73%) support the idea that pain medication should be given when the pain is quite bad, and 41.5% of respondents disagree that using pain medication for pain will lead to later drug abuse (Table 3).

**Table 3:** Response of parents for MAQ items (*n* = 102).

MAQ items	Agree, %	Disagree, %	Uncertain, %
1. Children should be given pain medication as little as possible because of side effects	61	22	17
2. Children who take pain medication for pain may learn to take drugs to solve other problems	26.8	29.3	43.9
3. Pain medication works the same no matter how often it is used	29.3	43.9	26.8
4. Pain medication works best when it is given as little as possible	63.4	24.4	12.2
5. Pain medication has many side effects	48.8	29.3	22
6. Children will become addicted to pain medication if they take it for pain	19.5	39	41.5
7. There is little need to worry about side effects from pain medication	43.9	22	34.1
8. It is unlikely a child will become addicted to pain medication if taken for pain	34.1	31.7	34.1
9. Pain medication is addictive	19.5	36.6	43.9
10. Pain medication works best if saved for when the pain is quite bad	73.2	17.1	9.8
11. Using pain medication for children's pain leads to later drug abuse	19.5	41.5	39
12. There is little risk of addiction when pain medication is given for pain	41.5	24.4	34.1
13. Children learn how to use pain medication responsibly when it is given for pain	34.1	46.3	19.5
14. Side effects are something to worry about when giving children pain medication	41.5	34.1	24.4
15. The less often children take pain medication for pain, the better the medicine works	48.8	31.7	19.5
16. Giving children pain medication for pain teaches proper use of drugs	34.1	41.5	24.4

### Analysis of factor structure

Exploratory factor analysis using maximum likelihood method with varimax rotation is used for analysing the factor structure and correlation between items included in the scale. Initially we did not get the desired result as some of items were loading on the other factor. We removed (item 3 from PPEP and 6, 15 from MAQ) those factors incrementally and get the following final results.

#### Parental pain expression perception (PPEP)

A factor analysis with Varimax rotation of the PPEP suggested a 3-factor solution, explaining 66% of the variance. The factors were: attention seeking (items 6, 7, and 9) explained 20.51% of the variance; play and tell parents (items 2, 4) explained 18.9% of the variance; and feeling and expressing pain (items 1, 5, and 8) explained 14.8% of the variance. The Cronbach's alpha, calculated for the whole measure, of 0.79 suggested internal consistency among the items (Table 4).

**Table 4:** Factors analysis of PPEP items.

PPEP items	Attention seeking	Play and tell parents	Feeling, expressing pain
6. Children exaggerate pain	0.77		
7. Children complain about pain to get attention	0.72		
9. Children in pain have trouble sleeping	0.65		
2. Children always tell their parents when they are in pain		0.86	
4. Children who are playing are not in pain		0.64	
5. Children experiencing pain report it immediately			0.76
8. Children feel pain less than adults			0.75
1. Children always express pain by crying or whining			0.87

#### Medication attitude questionnaire (MAQ)

MAQ factor analysis with Varimax rotation produced a 5-factor solution explaining 70.1% of the MAQ variance. The pain medication side effect (items 1, 9, 10, 11) explained 19.5% of the variance; avoidance of side effects (items 4, 12) explained 14.9% of the variance; less risk of side effects (items 3, 7, 8) explained 11.62% of the variance; high risk of side effects (items 5, 13, 14) explained 9.5% of the variance; and proper use of drugs (items 2, 16) explained 8.75% of the variance. To examine the internal consistency of the items, a Cronbach's alpha was calculated and found to be 0.68 (Table 5).

**Table 5:** Factor analysis of MAQ items.

MAQ items	Side effects	Avoidance	Less risk	High risk	Proper use
1. Children should be given pain medication as little as possible because of side effects	−0.65				
9. Pain medication is addictive	0.64				
10. Pain medication works best if saved for when the pain is quite bad	0.63				
11. Using pain medication for children's pain leads to later drug abuse	0.77				
4. Pain medication works best when it is given as little as possible		0.76			
12. There is little risk of addiction when pain medication is given for pain		0.77			
7. There is little need to worry about side effects from pain medication			0.54		
8. It is unlikely a child will become addicted to pain medication if taken for pain			0.84		
3. Pain medication works the same no matter how often it is used			0.695		
5. Pain medication has many side effects				0.81	
13. Children learn how to use pain medication responsibly when it is given for pain				0.56	
14. Side effects are something to worry about when giving children pain medication				0.62	
2. Children who take pain medication for pain may learn to take drugs to solve other problems					0.92
16. Giving children pain medication for pain teaches proper use of drugs					0.84

**Figure 1:**
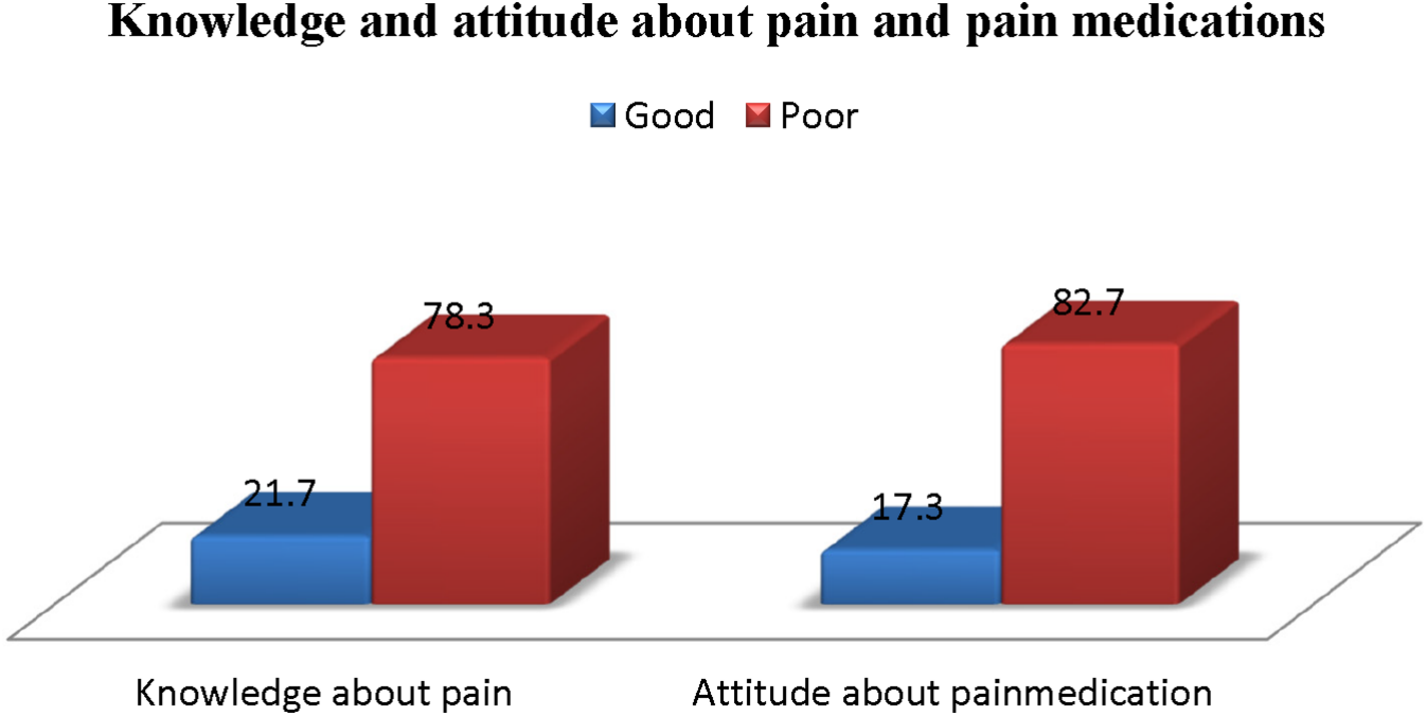
Parental knowledge and attitude about pain and pain medications.

From the total number of participants, only 21.7% of parents have good knowledge about pain (with a pain knowledge score of mean = 5.1, SD = 1.87), and 17.3% of parents have a good attitude about pain medication (with a medication attitude score of mean = 4.77, SD = 1.56) (Figure 1). In terms of sociodemographic categories, 17.7% of parents older than forty years old and 7.2% of parents between the ages of thirty and forty-nine have good knowledge. Furthermore, compared to fathers (9.3%), mothers (28.4%) had better awareness of Paediatric pain, according to our study.

### Predictors of parental knowledge and attitude of postoperative paediatric pain

Stepwise linear regression analysis was used to examine the impact of socio demographic variables of parents and children on PPEP and MAQ scores. The regression result for PPEP factors shows parents of younger children and parents from rural area are more likely to score higher in attention seeking sub-score of PPEP. Less educated parents are more likely to score higher in play, tell parents factors of PPEP, while parents from rural residence are more likely to perceive children express when they feel pain (Feeling, expressing pain). The regression result for MAQ factors shows parents from urban residence and those with employment status are more likely to perceive the side effects of pain medications (Side effects factors). Middle income parents and mothers scored less in avoidance factors of MAQ, while those parents who are employed are more likely to score higher in less risk factor. Less educated parents and parents from urban area are more likely to perceive the negative consequence of pain medications (high risk), while less educated parents scored higher on proper use of drug factors of MAQ (Table 6).

**Table 6:** Stepwise linear regression of the PPEP and MAQ sub-scores.

Variables	*β*	95%, CI	*P*-value
Stepwise regression of the PPEP sub-score (Attention seeking)			
Age of child	−0.43	(−0.78, −0.28)	0.001
Place of residence	−0.53	(−0.53, −0.23)	0.001
Educational level	0.4	(0.25, 0.77)	0.001
Stepwise regression of the PPEP sub-score (Play, tell parents)			
Educational level	−0.32	(−0.42, −0.073)	0.006
Relationship with parents	−0.3	(−0.69, −0.067)	0.018
Stepwise regression of the PPEP sub-score (Feeling, expressing pain)			
Place of residence	−0.29	(−0.54, −0.052)	0.017
Stepwise regression of the MAQ sub-score (Side effects)			
Educational level	−0.312	(−0.23, −0.05)	0.003
Place of residence	0.44	(0.19, 0.52)	0.001
Income	−0.25	(−0.38, −0.036)	0.019
Status of employment	0.474	(0.23, 0.54)	0.001
Stepwise regression of the MAQ sub-score (Avoidance)			
Income	−0.29	(−0.71, −0.094)	0.012
Relationship with the child	−0.46	(−0.88, −0.28)	0.001
Stepwise regression of the MAQ sub-score (Less risk)			
Status of employment	2.32	(0.03, 0.39)	0.022
Stepwise regression of the MAQ sub-score (High risk)			
Educational level	−3.04	(−0.36, −0.074)	0.003
Place of residence	2.73	(0.094, 0.59)	0.008
Stepwise regression of the MAQ sub-score (Proper use)			
Age of the mother	−0.39	(−0.47, −0.16)	0.001
Education level	−0.51	(−0.52, −0.22)	0.001
Income	0.38	(0.21, 0.72)	0.001

## Discussion

This study has used questionnaires (PPEP and MAQ) to assess parental knowledge and attitude about Paediatric pain and pain medication. The questionnaires were used for the first time in Ethiopia to assess parental knowledge and attitude about pain, with the data used as a baseline for further study. According to the result of our study, a significant number of parents hold poor knowledge about postoperative pain and the wrong attitude about pain medications. This significant knowledge and attitudinal barrier might be due to the higher cut-off point used (≥80%) for grouping parents having good or poor knowledge and attitude in this study. Research findings demonstrate that social, cultural, or religious factors significantly contribute to poor parental attitudes regarding children's pain management in Ethiopian context. A community-based cross-sectional study involving 267 parents of children under 18 revealed that, in the Tole District of South West Oromia, Ethiopia, 85.9% of parents utilised traditional medicine for their children (19).

A few studies conducted on parental knowledge and attitude about Paediatric pain have similar findings to our study results. A number of misconceptions concerning Paediatric pain management were discovered in a descriptive cross-sectional research of Saudi mothers to evaluate their attitudes regarding their children's pain and how it is managed. These erroneous beliefs may have an impact on the children' quality of life (13). Poor preoperative parental belief over analgesic administration for children have significantly affected postoperative analgesic administration to children for pain management (13). Another longitudinal study involving 132 parents of children who were aged 2–12 years reported, parents' responses have indicated significant barriers in the attitudinal survey about pain and pain medications (20).

Based on the PPEM parental score, nearly 80% of parents held poor knowledge about Paediatric pain in our study. Believing children always express pain by whining or crying, agreeing with children that playing is not in pain, considering children immediately tell their parents when they are in pain, and coming to an agreement with the thought that children are not in pain were the identified items with significant misconceptions about knowledge of Paediatric pain. This finding is consistent with previous studies conducted to assess parental knowledge and attitude about pain in hospitalized children, although the level of misconception was significantly higher in our study. A descriptive correlational study demonstrates parents displayed moderate levels of knowledge (9) and another study conducted to explore parental attitudes toward children's pain and analgesic drugs showed a considerable proportion of parents held misconceptions about how children express pain (21).

Among sociodemographic subgroups, 32.2% of parents under the age of 20–29 years have good knowledge, followed by 7.2% between 30 and 39 years and 17.7% aged ≥40 years. A similar study conducted is congruent with our study result, even though the figure is much lower in our study, which might be related to the cut-off point used (13, 22). Our study also found that the percentage of mothers with good knowledge (28.4%) about paediatric pain was higher than that of fathers (9.3%). Our study findings are consistent with a number of cross-sectional studies conducted previously to evaluate the level of knowledge about paediatric pain among parents (20, 23). This result might be related since mothers are the primary caregivers for their children. Regarding their place of residence, 34.3% of parents from urban areas understood paediatric pain better, whereas only 3.5% of parents from rural areas had good knowledge. This result matches previous studies (9) conducted, which reported that mothers who participate in the study have a higher percentage of knowledge about postoperative paediatric pain compared to fathers.

As shown in Table 3, parents hold multiple attitudinal misconceptions about pain medication for their children. For example, 61% of parents agreed children should be given pain medication as little as possible because of side effects. Study conducted in the United Kingdom (10) reported similar findings with our study result, although the proportion of parents with the misconception is higher in our study. A large proportion of parents (78%) perceive that pain medication works best if saved for when the pain is quite bad. This misconception is supported by previous studies conducted to evaluate the attitude towards pain and analgesics among Korean parents (21). Parents hold an erroneous attitude about the dosage of pain medications; nearly 64% of parents agreed that pain medication works best when it is given as little as possible.

One of the barriers to effective management of paediatric pain is fear of side effects and addiction to pain medications (24). This thought was also significantly observed in our study: 19.5% of parents believe pain medication is always addictive, and children will become addicted to pain medication if they take it for pain. 48.8% of parents consider the less often children take pain medication for pain, the better the medicine works. Other studies have reported similar results regarding beliefs in such myths and the resultant negative attitude towards pain medication among parents of different backgrounds (25).

This study explored predictors of parental knowledge and attitude toward children's pain expression. According to our results, less educated parents believe that children always express their feelings when they are in pain. In line with this finding, parents with low education levels scored higher on the Active, Loud behaviours factor of the PPEP (25). In the present study, parents of younger children consider children exaggerate and complain about pain compared to parents of older children. This is consistent with the previous study indicating parents of younger children agreed children experience the fewest pain but receive highest pain and distress estimates compared to parents of older children (26).

In addition to parents' perceptions of pain, this study also explored their perceptions of pain medications. In terms of predicting attitudes toward pain medication, the previous study indicates that less educated parents scored higher in the avoidance factors of MAQ (25), However, level of education did not have an impact, according to the findings of the present study. Another study conducted to evaluate parental perceptions about the dose of pain medication demonstrates that parents of older children have misconceptions about the dose of pain medication needed to treat children's pain (23), while the age of the child in our study did not have an impact on parental perceptions about the dose of pain medication needed.

## Strength and limitation of the study

To our knowledge, no studies in Ethiopia report on the parental knowledge and attitude towards Paediatric pain and pain medications. This study addressed a current and essential clinical problem that remains poorly managed by parents. This study is conducted on a single setting additional multi-centre studies with a larger sample are recommended. It is not known to what extent the study respondents were responsible for their child's pain management at home. Although the research team arranged for the questionnaires to be translated into the local language, no measures were taken to ensure cultural relevance of the measures.

## Conclusion

The overall knowledge and attitude of parents about postoperative pain and pain medication is poor. This misconception could affect effective Paediatric pain management. There should be an awareness-creation program about pain and pain medications, which will enhance parental knowledge and attitude.

## Data Availability

The original contributions presented in the study are included in the article/Supplementary Material, further inquiries can be directed to the corresponding author.
